# Leflunomide combined with low-dose prednisone inhibits proinflammatory T cells responses in myasthenia gravis patients

**DOI:** 10.3389/fneur.2022.961628

**Published:** 2022-09-09

**Authors:** Xin Huang, Hao Ran, Yingkai Li, Qian Ma, Changyi Ou, Li Qiu, Huiyu Feng, Weibin Liu

**Affiliations:** ^1^Department of Neurology, The First Affiliated Hospital, Sun Yat-sen University, Guangzhou, China; ^2^Guangdong Provincial Key Laboratory of Diagnosis and Treatment of Major Neurological Diseases, National Key Clinical Department and Key Discipline of Neurology, Guangzhou, China; ^3^School of Pharmaceutical Sciences, Sun Yat-sen University, Guangzhou, China

**Keywords:** myasthenia gravis, leflunomide, low dose prednisone, Th1 cells, Th2 cells, Th17 cells, Th9 cells

## Abstract

We previously found that leflunomide combined with low-dose prednisone rapidly improved the clinical symptoms of myasthenia gravis (MG), but we had not investigated the mechanism of this phenomenon. This study documents the effect of leflunomide combined with low-dose prednisone on pro-inflammatory T cells in MG patients. We compared 32 treated MG patients with 18 controls. We collected peripheral blood before treatment and 4, 8, and 12 weeks after treatment. We extracted peripheral blood mononuclear cells (PBMCs) and stimulated them with phorbol 12-myristate 13-acetate (PMA) + ionomycin and quantified IFN-γ, IL-4, IL-17, and IL-9 secretion through ELISA. We quantified T helper (Th) cells Th1 (CD3+CD4+IFN-γ+), Th2 (CD3+CD4+IL-4+), Th17 (CD3+CD4+IL-17A+) and Th9 (CD3+CD4+IL-9+) among PBMCs. The treatment significantly reduced IL-17 and IL-9 secretion in peripheral blood but did not affect IFN-γ levels. Significant decreases in IL-17 and IL-9 appeared at week 12, and the trend of change was similar to that of the MG composite score. Flow cytometry indicated that leflunomide combined with low-dose prednisone significantly reduced the frequency of Th1 and Th17 cells. These findings demonstrate the potential of this treatment as an alternative immunosuppressive therapy for MG.

## Introduction

Myasthenia gravis (MG) is an autoimmune disorder that affects the neuromuscular junction of the skeletal muscle. Corticosteroids are the first-line treatment for immune regulation of MG. In the long-term follow-up of a retrospective study on adolescent MG patients admitted to our hospital in the past 17 years, we observed that 64.1% of MG patients taking low-dose prednisone (≤ 0.25 mg/kg/day) experienced notable effects (1). It also showed that low-dose prednisone alone was ineffective in some patients. However, combining low-dose prednisone and immunosuppressive agents can markedly improve the efficacy and avoid the side effects of high-dose prednisone. Thus, clinicians should consider adding immunosuppressants in these cases.

Leflunomide is an isoxazole immunosuppressant that inhibits T and B cells proliferation. Leflunomide can inhibit the production and secretion of antibodies, and the expression of inflammatory cytokines, such as IL-1, IL-6, IL-17, TNF-α (2). Leflunomide is commonly used in the treatment of adults and children rheumatoid arthritis (3). Our previous studies have demonstrated that the immunosuppressant leflunomide is effective against myasthenia gravis. In 2016, we followed 15 prednisone-dependent MG patients treated with leflunomide (20 mg/day). After 6 months, 12 (80%) patients exhibited improved symptoms, and 11 of them showed quantitative myasthenia gravis (QMG) score reductions of more than three points, indicating major symptom improvement. In addition, the average daily dose of prednisone used by these patients was reduced from 24.3 to 12.3 mg (4). Next, we investigated the clinical effects of leflunomide combined with low-dose prednisone in 32 MG patients. The median MG composite (MGC) score dropped from 8.5 (at the time of enrollment) to 4 (after 12 weeks) (5).

However, the mechanism of action of leflunomide combined with corticosteroid in the treatment of MG remains unclear. Although MG is an autoimmune disease mediated by antibodies, its pathogenesis is closely related to cellular immunity, especially CD4 T-helper (Th) cells (6, 7). These cells have clear roles in immune response induction and B-cell function support (8). The present study documents the effects of leflunomide combined with low-dose prednisone on Th cells in the treatment of MG.

## Materials and methods

### Study population and controls

We enrolled patients from November 2017 to August 2019 at the Department of Neurology, First Affiliated Hospital of Sun Yat-sen University, China. The inclusion criteria were: clear clinical MG diagnosis (Morbid fatigue of affected skeletal muscle, meeting any one of the following 3 conditions: a. AChR-Ab/Musk-Ab positive; b. Neostigmine test positive; c. Low frequency repetitive electrical stimulation positive), age ≥15 years, MGC score ≥3 before treatment, and no use of corticosteroids and other immunosuppressants for the prior 3 months. The exclusion criteria were: leflunomide contraindication (based on investigator evaluation), history of infection and antibiotics use within the previous 2 weeks, history of HIV infection preventing immunosuppressants use, other autoimmune diseases, tuberculosis, hepatitis, or other infectious diseases, or severe heart, brain, and kidney diseases. Control subjects were matched for age and gender as closely as possible, were not receiving therapy for any chronic disease, and had no autoimmune disease history.

The treatment and healthy controls groups included 32 and 18 patients, respectively. The Ethics Committee of the First Affiliated Hospital of Sun Yat-sen University approved this study, and all the selected participants provided informed consent.

### Samples collection

The patients received 0.25 mg/kg/day of prednisone and 20 mg/day of leflunomide. At enrollment, we recorded demographic information, including gender, age-onset, onset symptoms, duration of disease, serum auto-antibody status, MGFA (myasthenia gravis foundation of America) classification, and thymectomy. In addition, we evaluated the MGC score, QMG score, and activities of daily living score at each visit. Comparison of MGC scores before and after 12 weeks of treatment was used to determine treatment efficacy. Specifically, after 12 weeks of treatment, improvement in MGC scores ≥ 3 points are considered responsive.

Before treatment and after 4, 8, and 12 weeks of treatment, we collected 15 mL peripheral blood samples in EDTA tubes. We separated peripheral blood mononuclear cells (PBMCs) by Ficoll density gradient centrifugation. Next, we placed the cells in a CoolCell cell freezing container with a cryopreservation solution (90% fetal bovine serum (FBS) and 10% dimethyl sulfoxide) and froze them in a −80 °C refrigerator. After 24 h, we transferred the cells to a liquid nitrogen tank for storage.

### Cell recovery

We took out the frozen cells from the liquid nitrogen tank and quickly thawed them into a 37°C water bath. Next, we washed them twice with RPMI medium containing 10% FBS (R10). Finally, we stained the cells with Trypan blue, counted them under a microscope, and adjusted their concentration to 1.5 × 10^6^ cells/mL by adding R10.

### ELISA

#### Cell culture

We loaded the PBMCs into two EP tubes, 700 μL/tube. We suspended the cells in culture medium alone (blank control) or mixed with a cell stimulant (phorbol 12-myristate 13-acetate [PMA]/ionomycin, [BD Biosciences, San Jose, CA, USA], 1:500) and then seeded them into a 96-well culture plate (200 μL/ well). We used two wells for each sample and culture condition.

#### ELISA test

We incubated the cells for 48 h at 37°C in 5% CO_2_ in a humidified incubator. We then collected the cell culture supernatants and quantified interferon (IFN)-γ, interleukin (IL)-4, IL-17, and IL-9 by ELISA. Our previous experiments confirmed that IFN-γ, IL-17, and IL-9 secretion peaks at 48 h, while IL-4 secretion peaks at 24 h. Therefore, we performed ELISA for IL-4 after 24 h of incubation and the rest after 48 h. We used ELISA kits (R&D Systems, MN, USA) according to the manufacturer's recommendations.

### Flow cytometry

#### Cell culture

After thawing cryopreserved cells, we mixed 500 μL of cell suspension with 1 μL of cell stimulant. We then incubated the cells for 5 h at 37°C in 5% CO_2_ in a humidified incubator before performing flow cytometry.

#### Flow cytometry

We washed the cultured cells with washing buffer. Then, we stained the cells with 50 μL of LIVE/DEAD-eFlour505 (eBioscience, San Diego, CA, USA) in phosphate-buffered saline for 15 min at room temperature to remove dead cells. Next, we stained cell surface antigens with a cocktail consisting of titrated volumes of anti-CD3 PE-Dazzle and anti-CD4-FITC for 15 min at room temperature. After cell surface staining, we treated the cells with cytofix/cytoperm (eBioscience) according to the manufacturer's recommendations. We then performed intracellular staining for 20 min at room temperature using the following conjugates: anti-IFN-γ APC, anti-IL-17A APC, and anti-IL-9 PE. We used the homotypic controls of these cytokines as controls. These fluorescent antibodies were all purchased from Biolegend (San Diego, CA, USA). Finally, we fixed the cells with 1% paraformaldehyde and acquired data on a CytoFLEX S flow cytometer (Beckman Coulter, Brea, FL, USA) using compenbeads when needed. After the test, we analyzed the data with FlowJo 10.0 (Tree Star) software.

### Statistical analysis

We analyzed data using SPSS for Windows 22.0 (IBM, Armonk, NY, USA). We compared the groups using Welch's corrected unpaired t/rank sum test, compared each group before and after treatment using paired t/rank sum test. *P*-values < 0.05 were considered statistically significant. Graphs were generated using GraphPad Prism 6 software (Graphpad, La Jolla, CA, USA).

## Results

### Clinical effect

**Figure 1:**
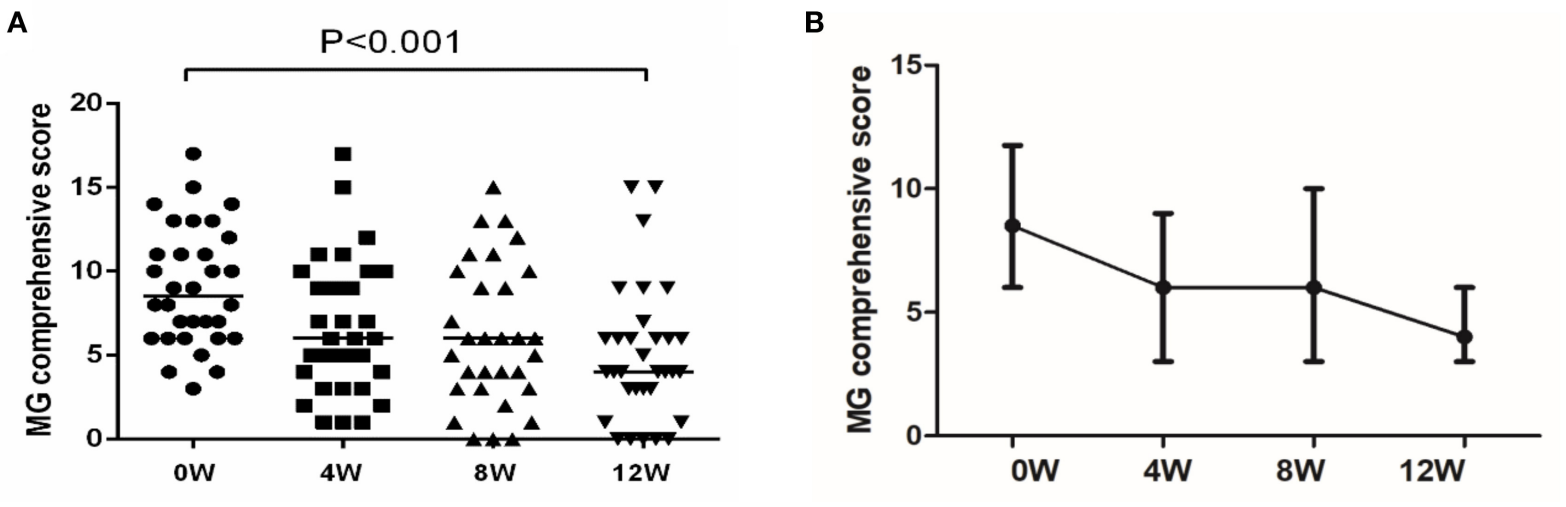
Changes in MGC during treatment with leflunomide combined with low-dose prednisone. **(A)** MGC at each follow-up time point. Reproduced with permission from Huang et al (5). **(B)** Changes in MGC. The MGC was a continuous variable with a non-normal distribution; we compared the values using a paired rank sum test.

Table 1 details the basic information and clinical characteristics of the 32 included MG patients. The male to female ratio was 1:1. At the 12-week follow-up, 59.4% of the patients (19/32) experienced improvements (MGC score decreased by at least three points). Before enrollment, the median MGC score was 8.5, and it dropped to 4 at the 12-week follow-up (Figure 1A). As Figure 1B shows, the MGC score changed as soon as week 4 (first follow-up). Then it did not significantly change at week 8, but it drastically dropped at week 12. Thymectomy was previously performed in 19 patients; none had a thymoma. Of the 19 patients who underwent thymectomy, 11 were effective after 12 weeks of medication. Of the 13 patients who did not have thymectomy, 8 were effective. There was no significant difference in effectiveness between the two groups (χ2 test, *P* = 0.837) (5).

**Table 1:** Basic information for the patients treated with leflunomide and low-dose prednisone.

**Group**	**no**.	**Sex**	**Age (years)**	**Duration** **(years)**	**AChR-Ab titer (nmol/L)**	**MGFA** **cl classification**	**Thymectomy (Yes/No)**	**QMG**	**ADL**	**MGC**
								**Before treatment**		
MG patients group	1	F	19	3	18.08	IIa	No	13	6	10
	2	F	28	0.2	38.02	IIIa	No	15	6	14
	3	F	39	8	0.61	IIa	No	3	4	3
	4	F	37	9	0.69	I	No	6	7	6
	5	M	23	3	0.66	I	No	6	6	6
	6	F	35	1	28.76	IIa	No	9	6	7
	7	M	52	0.2	0.29	I	No	7	6	6
	8	F	18	1	1.34	IIa	No	7	3	7
	9	M	57	1	0.40	IIa	No	9	5	11
	10	M	66	1	0.47	I	No	6	6	6
	11	F	36	31	3.19	IIa	No	8	7	8
	12	M	19	0.4	31.93	I	No	7	5	5
	13	F	20	0.7	39.82	IIa	No	8	4	7
	14	M	20	1	39.23	IIa	Yes	11	4	8
	15	M	37	0.4	31.10	IIIa	Yes	12	8	8
	16	M	25	14	0.98	I	Yes	8	6	6
	17	F	19	9	0.40	IIa	Yes	13	6	10
	18	M	15	1	8.13	IIa	Yes	6	4	4
	19	F	16	12	4.78	IIa	Yes	10	9	7
	20	M	25	0.4	0.86	IIa	Yes	14	7	9
	21	F	39	1	36.59	IIb	Yes	17	9	13
	22	F	30	3	3.37	IIIa	Yes	14	5	14
	23	M	31	15	0.21	I	Yes	4	4	4
	24	F	30	0.8	0.43	IIa	Yes	13	7	11
	25	M	62	0.3	10.14	IIIa	Yes	16	8	13
	26	M	24	2	23.17	IIb	Yes	17	7	13
	27	F	50	12	38.08	IIa	Yes	12	7	11
	28	M	32	0.6	41.35	IIb	Yes	21	12	17
	29	M	51	25	1.00	IIa	Yes	11	8	10
	30	M	15	5	25.73	IIb	Yes	19	9	15
	31	F	48	0.5	9.17	IIa	Yes	12	10	12
	32	F	28	20	0.58	IIa	Yes	11	6	9
Controls	1	M	30	-	-	-	-	-	-	-
	2	M	47	-	-	-	-	-	-	-
	3	M	40	-	-	-	-	-	-	-
	4	F	44	-	-	-	-	-	-	-
	5	M	26	-	-	-	-	-	-	-
	6	F	50	-	-	-	-	-	-	-
	7	F	43	-	-	-	-	-	-	-
	8	F	22	-	-	-	-	-	-	-
	9	M	46	-	-	-	-	-	-	-
	10	F	22	-	-	-	-	-	-	-
	11	M	42	-	-	-	-	-	-	-
	12	M	39	-	-	-	-	-	-	-
	13	F	22	-	-	-	-	-	-	-
	14	F	27	-	-	-	-	-	-	-
	15	M	35	-	-	-	-	-	-	-
	16	F	45	-	-	-	-	-	-	-
	17	M	42	-	-	-	-	-	-	-
	18	F	22	-	-	-	-	-	-	-

### Leflunomide combined with low-dose prednisone can significantly inhibit the secretion of IL-17 and IL-9 in PBMCs of MG patients

**Figure 2:**
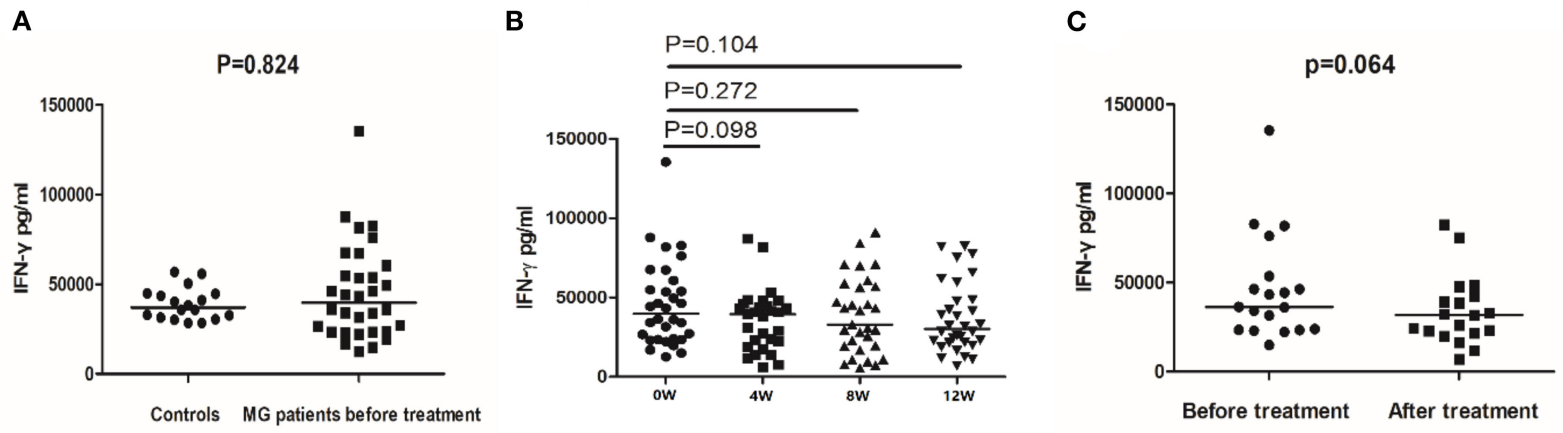
IFN-γ secretion detected by ELISA in controls and MG patients. **(A)** IFN-γ secretion in PBMCs of healthy controls and MG patients before treatment. **(B)** IFN-γ secretion in PBMCs of MG patients before treatment and after 4, 8, and 12 weeks of treatment. **(C)** IFN-γ secretion in PBMCs of responsive MG patients before treatment and at 12 weeks. The horizontal bars indicate the median. The amount of IFN-γ secretion was a continuous variable with a non-normal distribution. We compare MG patients and controls using Welch's corrected unpaired rank sum test. We compared before and after treatment using a paired rank sum test.

We collected PBMCs before and after 4, 8, and 12 weeks of leflunomide combined with low-dose prednisone treatment. Next, we stimulated the cells (PMA/ionomycin) and quantified IFN-γ secretion by ELISA. Before treatment, the median IFN-γ secretion was 39789.08 pg/mL in MG patients and 37061.84 pg/mL in healthy controls. IFN-γ secretion was similar in healthy controls, untreated MG patients (Figure 2A), and treated MG patients (Figure 2B). The clinical data indicated that 19 treated patients experienced alleviated symptoms. However, the treatment did not affect IFN-γ secretion in stimulated PBMCs from these responsive patients (Figure 2C).

**Figure 3:**
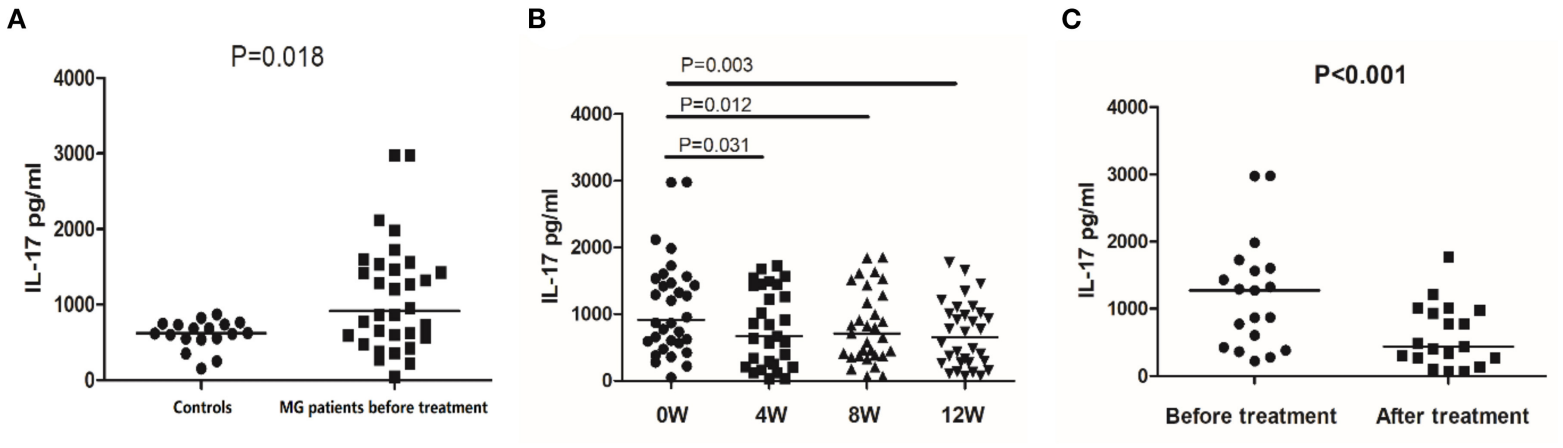
IL-17 secretion detected by ELISA in controls and MG patients. **(A)** IL-17 secretion in PBMCs of healthy controls and MG patients before treatment. **(B)** IL-17 secretion in PBMCs of MG patients before treatment and after 4, 8, and 12 weeks of treatment. **(C)** IL-17 secretion in PBMCs of responsive MG patients before treatment and at 12 weeks. The horizontal bars indicate the median. The amount of IL-17 secretion was a continuous variable with a non-normal distribution; We compare MG patients and controls using Welch's corrected unpaired rank sum test. We compared before and after treatment using a paired rank sum test.

Before treatment, the median secretion of IL-17 was 913.79 pg/mL in MG patients and 621.60 pg/mL in healthy controls (*P* = 0.018, Figure 3A). After 12 weeks of treatment, the IL-17 median secretion of MG patients was 653.83 pg/mL, comparable to that of the healthy controls (*P* = 0.693). Figure 3B shows the IL-17 secretion over the 12 weeks of treatment. In the 19 responsive patients, the median secretion of IL-17 after treatment was 438.44 pg/mL, significantly lower than before treatment 1275.22 pg/mL (*P* < 0.001) (Figure 3C). In the 13 unresponsive patients, treatment did not affect IL-17 secretion (*P* = 0.561).

**Figure 4:**
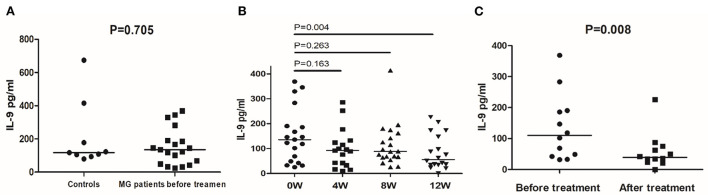
IL-9 secretion detected by ELISA in controls and MG patients. **(A)** IL-9 secretion in PBMCs of healthy controls and MG patients before treatment. **(B)** IL-9 secretion in PBMCs of MG patients before treatment and after 4, 8, and 12 weeks of treatment. **(C)** IL-9 secretion in PBMCs of responsive MG patients before treatment and at 12 weeks. The horizontal bars indicate the median. The amount of IL-9 secretion was a continuous variable with a non-normal distribution; We compare MG patients and controls using Welch's corrected unpaired rank sum test. We compared before and after treatment using a paired rank sum test.

Next, we quantified IL-9 secretion in stimulated (48 h) PBMCs from 17 MG patients and 9 controls through ELISA. Before treatment, patients and healthy controls had similar peripheral blood IL-9 secretion levels (Figure 4A). After 12 weeks, the treatment reduced the median secretion of IL-9 in MG patients to 50.53 pg/mL (*P* = 0.004, compared with untreated MG patients, Figure 4B). Next, we looked at responsive and unresponsive patients separately. In 12 responsive patients, the treatment significantly reduced the median secretion of IL-9 (39.31 pg/mL *vs*. 110.26 pg/mL, *P* = 0.008, Figure 4C). However, in unresponsive patients, the treatment did not affect IL-9 secretion (*P* = 0.225).

Finally, we quantified IL-4 secretion in stimulated (24 h) PBMCs from 17 MG patients and 9 healthy controls. Before treatment, the median secretion of IL-4 was 32.09pg/mL in MG patients and 34.88pg/mL in healthy controls (*P* = 0.172). After 12 weeks of treatment, the IL-4 median secretion of MG patients was 18.649pg/mL. The treatment did not affect IL-4 secretion (*P* = 0.496).

### IFN-Γ, IL-17, IL-9, and IL-4 changes in PBMCs of responsive MG patients

**Figure 5:**
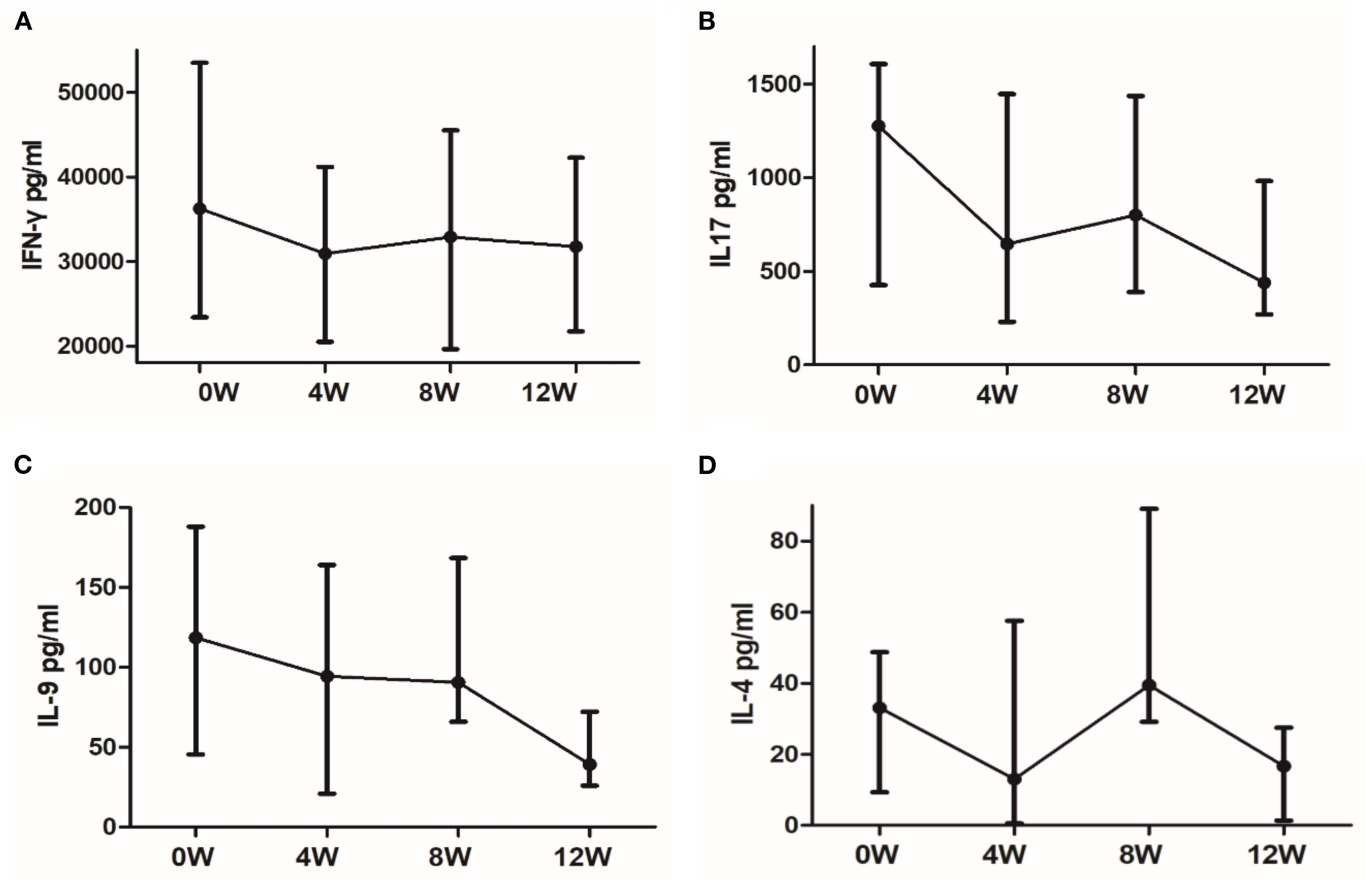
Cytokines secretion trends in responsive patients. **(A)** IFN-γ, **(B)** IL-17, **(C)** IL-9, and **(D)** IL-4 secretion trends. The dots indicate the median. The horizontal bars indicate the quartiles.

We plotted the median cytokines secretion in responsive patients over time and found that each cytokine had a distinct trend (Figure 5). As Figure 5A shows, the secretion of IFN-γ is significantly reduced in the first 4 weeks. Meanwhile, in responsive patients, IL-17 secretion decreased significantly in the first 4 weeks and decreased again at 12 weeks (Figure 5B). This trend is consistent with the changes in MGC score. However, IL-9 secretion did not drop until week 12 (Figure 5C). Finally, IL-4 secretion had no specific trend (Figure 5D).

### Leflunomide combined with low-dose prednisone can decrease frequency of Th1 and Th2 cells in MG patients

**Figure 6:**
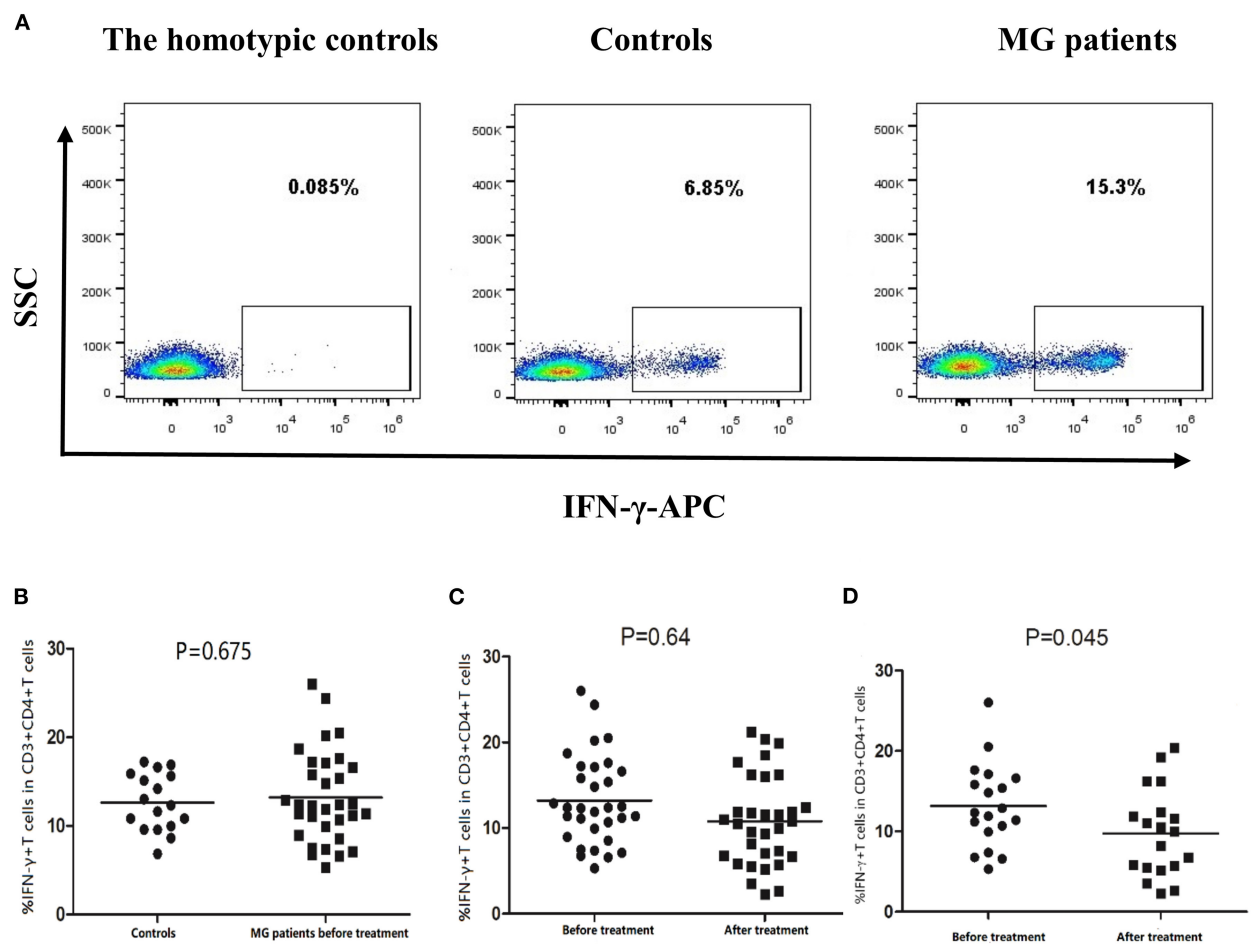
Frequency of Th1 cells (CD3+CD4+IFN-γ+ T cells) in PBMCs of controls and MG patients. **(A)** Gating on CD3+CD4+ T cells, intracellular cytokine analysis of IFN-γ on PBMCs of controls and MG patients after cell culture. **(B)** Th1 cell frequency in controls and MG patients before treatment. **(C)** Th1 cell frequency in MG patients before and after treatment. **(D)** Th1 frequency in responsive patients before and after treatment. The horizontal bars indicate the mean. We compare MG patients and controls using unpaired *t* test. We compared before and after treatment using paired *t*–test.

**Figure 7:**
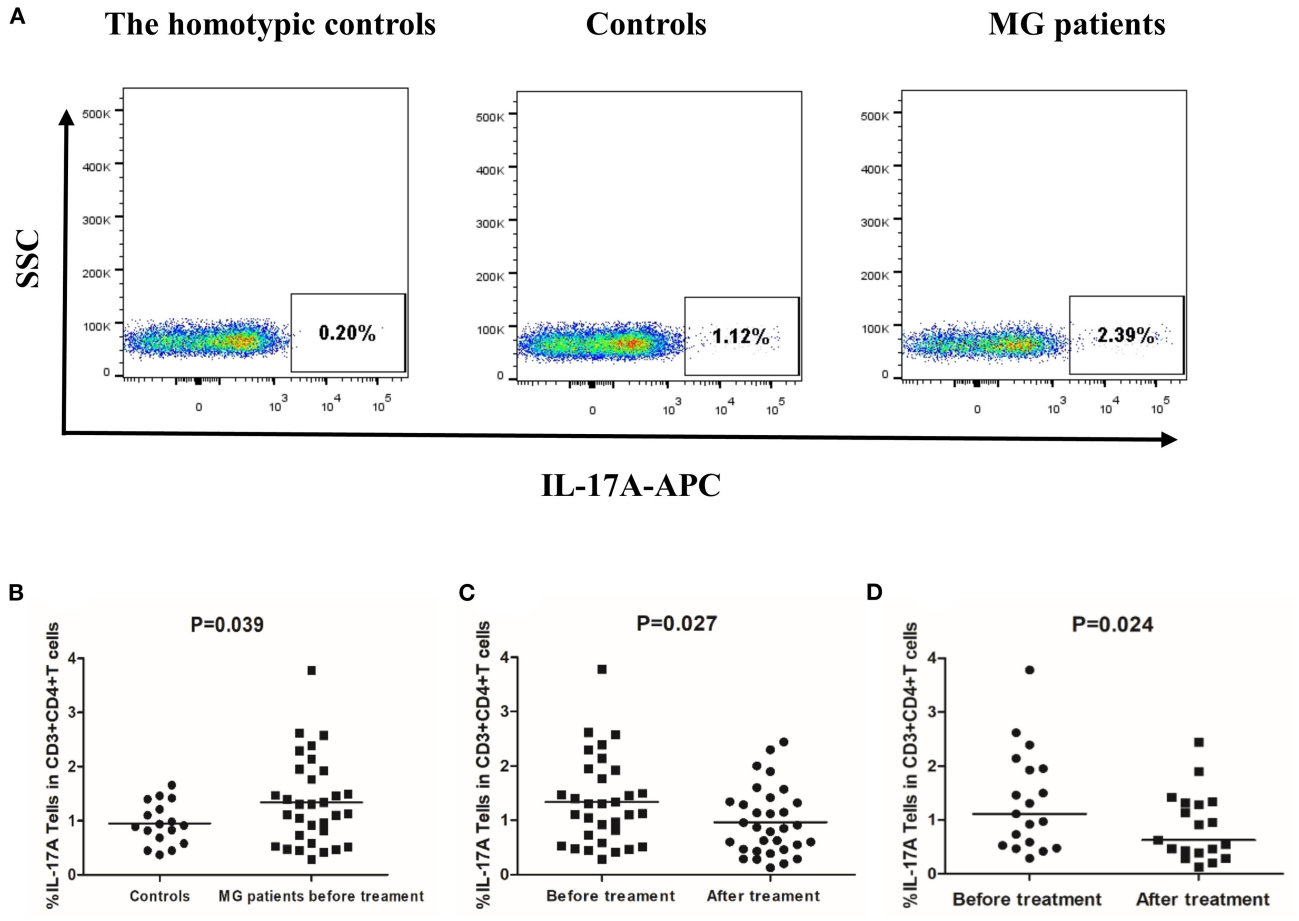
Frequency of Th17 cells (CD3+CD4+IL-17A+ T cells) in PBMCs of controls and MG patients. **(A)** Gating on CD3+CD4+ T cells and intracellular cytokine analysis for IL-17A on PBMCs of controls and MG patients after cell culture. **(B)** Th17 cell frequency in controls and MG patients before treatment. **(C)** Th17 cell frequency in MG patients before and after treatment. **(D)** Th17 frequency in responsive patients before and after treatment. The horizontal bars indicate the mean. We compare MG patients and controls using unpaired *t*–test. We compared before and after treatment using paired *t*-test.

The ELISA results confirmed that the leflunomide combined with low-dose prednisone treatment affected IL-17 and IL-9 secretion, especially in the responsive patients. Next, we confirmed the effect of this treatment on Th cells by performing flow cytometry on PBMCs of MG patients (Figures 6A, 7A).

The mean frequency of Th1 cells was 14.47% ± 4.39% in MG patients before treatment and 12.63% ± 3.22% in healthy controls. The difference was not statistically significant (Figure 6B). Moreover, the mean frequency of Th1 cells in MG patients treated for 12 weeks was 10.79% ± 5.25%, which was not statistically different from before treatment (Figure 6C). However, in the responsive patients, the mean frequency of Th1 cells decreased significantly after treatment (*P* = 0.045, Figure 6D).

The mean frequency of Th17 cells was 1.55% ± 0.86% in MG patients before treatment and 0.95% ± 0.38% in healthy controls (*P* = 0.039, Figure 7B). After 12 weeks of treatment, the frequency of Th17 cells was significantly lower than before treatment (*P* = 0.027, Figure 7C). However, not all patients experienced this effect. In responsive patients, the frequency of Th17 decreased significantly after treatment (*P* = 0.024, Figure 7D). In unresponsive patients, treatment did not significantly affect Th17 cells frequency (*P* = 0.237).

The median frequency of Th2 cells was 1.87% (1.17%, 2.50%) in MG patients before treatment and 2.25%(1.54%, 3.84%)in healthy controls (*P* = 0.147). After 12 weeks of treatment, The median frequency of Th2 cells was 1.49% (0.94%, 2.35%). After 12 weeks of treatment, the frequency of Th2 cells was significantly lower than before treatment (*P* = 0.011).

MG patients and healthy controls had comparable Th9 cells frequencies. In addition, leflunomide combined with low-dose prednisone did not significantly change the frequency of Th9 cells in MG patients, even responsive ones.

## Discussion

According to the MG treatment guidelines, corticosteroids are the first-line drugs. Their efficacy reaches 80–90% (9). However, long-term or high-dose use of corticosteroids can cause serious side effects such as infections, high blood sugar, high blood pressure, and osteoporosis. Therefore, many patients cannot use high-dose corticosteroids for extended periods. Meanwhile, low doses of corticosteroids are less effective (1), but combining them with other immunosuppressive agents can yield better results and fewer side effects. For example, leflunomide, an isoxazole immunosuppressive agent with anti-proliferative activity, can inhibit both cellular and humoral immunity (10). Our previous studies have confirmed that leflunomide combined with low-dose prednisone can effectively treat MG with few side effects (2). In this study, we explored the immunological effects of this combination on pro-inflammatory T cells response in MG patients.

Th cells assist B cells to produce antibodies and promote the differentiation and maturation of other T cells by secreting various cytokines, such as IFN-γ (mainly secreted by Th1 cells). Studies have confirmed that MG patients have significantly higher IFN-γ levels than healthy people and that IFN-γ levels decreased after symptoms improved (11). However, in our study, untreated MG patients and healthy controls had similar IFN-γ secretion levels. The fact that 19 MG patients (out of the 32 recruited) had undergone thymectomy may account for this discrepancy since we previously observed that thymectomy significantly reduced IFN-γ secretion (results not shown). Although the ELISA results indicated that the treatment did not affect IFN-γ secretion in MG patients, the flow cytometry results confirmed that it reduced IFN-γ secretion in responsive patients by inhibiting Th1 cells. IFN-γ is mainly secreted by Th1 cells, but NK cells and DC cells also secrete some IFN-γ. However, we speculate that leflunomide combined with low-dose prednisone does not significantly inhibit these cells.

Th17 cells play an essential regulatory role in autoimmune diseases (12). IL-17 is mainly secreted by Th17 cells, which are closely related to asthma and systemic lupus erythematosus (13, 14). In 2008, Baggi showed that IgG immunoadsorption decreased IL-17 and alleviated symptoms in MG patients (15), confirming that IL-17 is related to the occurrence of MG. Another study found that AChR-Ab levels in MG patients are positively correlated with Th17 cells, and the frequency of Th17 cells is correlated with the QMG score (16). However, few immunosuppressants can inhibit Th17 cells. Th17 are resistant to corticosteroids (17). Tacrolimus inhibits Th17 cells, albeit only weakly (18). Our research confirms that leflunomide combined with low-dose prednisone can significantly reduce IL-17 secretion and inhibit Th17 cells. This treatment may be a new option for MG patients who do not respond well to other immunosuppressive agents.

In 2008, a new type of Th cell, characterized by the secretion of IL-9, was discovered and named Th9 (19). Several studies have confirmed that Th9 cells are associated with many autoimmune diseases and with disease severity. In the chronic phase of experimental autoimmune MG, the frequency of Th9 cells is dramatically high, and EAMG rats treated with anti-IL-9 antibodies showed improvement in clinical symptoms and a significant decrease in AChR antibody levels (20). Yao latest study found that rats immunized with AChR97-116 peptide were injected with exogenous recombinant rat IL-9(RR IL-9), which had the same results as those in previous studies (21). In addition, the pathogenic role of Th9 cells and IL-9 in MG patients has also been confirmed recently. The levels of Th9 and IL-9 in peripheral blood of MG patients are significantly increased, and are positively correlated with the titer of AChR antibody in patients' serum (22). These studies suggest that Th9 cell subsets and IL-9 play an important role in the pathogenesis of MG and EAMG. Inhibition of Th9 is expected to be a new treatment method for MG. Our study confirms that leflunomide combined with low-dose prednisone can significantly reduce IL-9 secretion, especially in responsive patients. Unfortunately, the frequency of Th9 cells in CD4 T cells is very low, making flow cytometric detection difficult. In the future, we aim to improve the detection method and then compare Th9 cells frequencies in patients and healthy people.

IL-4, mainly secreted by Th2 cells, regulates the production of Ig G1 and mediates humoral immunity. Previous studies have found that IL-4 may be directly involved in immune tolerance or participate in immune response regulation by promoting cell secretion function, and may play a protective role in the pathogenesis of EAMG (23). However, the role of IL-4 in MG remains unclear. One study reported that serum and lymphocyte culture supernatant IL-4 concentrations in MG patients were not significantly different from healthy controls (24), while another study reported that serum IL-4 concentrations in MG patients were lower than those in healthy controls (25). Our study found MG patients and healthy controls had similar IL-4 secretion levels. We speculate that IL-4 or Th2 cells may not be directly involved in the pathogenesis of MG. The imbalance of Th1/Th2 cells may be the more important mechanism.

This study demonstrates that leflunomide combined with low-dose prednisone can reduce IL-17 and IL-9 secretion in the peripheral blood of MG patients. It can also inhibit Th1, Th2 and Th17 cells. Additionally, Th17 cells seemed particularly sensitive to this treatment, which is noteworthy since few immunosuppressants can inhibit Th17 cells. These findings show the potential of leflunomide combined with low-dose prednisone as an alternative immunosuppressive therapy for MG.

## Data availability statement

The original contributions presented in the study are included in the article/supplementary material, further inquiries can be directed to the corresponding authors.

## Ethics statement

The studies involving human participants were reviewed and approved by the Ethics Committee of the first affiliated hospital of Sun Yat-sen University. Written informed consent to participate in this study was provided by the participants' legal guardian/next of kin.

## Author contributions

XH participated in patient recruitment, flow cytometry and ELISA experiments, statistical analysis, and manuscript writing and submission. HR contributed to database development and data analysis. YL contributed to data processing, manuscript writing, and revision. QM contributed to sample collection and ELISA experiments. CO participated in the treatment of some patients and contributed to data collection. LQ contributed to data collection. HF established the database. WL designed and supervised the experiments, analyzed data, and contributed to manuscript writing. All authors contributed to the article and approved the submitted version.

## Funding

This study was supported by grants from the GuangDong Basic and Applied Basic Research Foundation (2020A1515110909), Chinese NSF (81620108010), Guangdong Provincial Key Laboratory of Diagnosis and Treatment of Major Neurological Diseases (2020B1212060017), Guangdong Provincial Clinical Research Center for Neurological Diseases (2020B1111170002), Southern China International Joint Research Center for Early Intervention and Functional Rehabilitation of Neurological Diseases (2015B050501003 and 2020A0505020004), Guangdong Provincial Engineering Center for Major Neurological Disease Treatment, Guangdong Provincial Translational Medicine Innovation Platform for Diagnosis and Treatment of Major Neurological Disease, and Guangzhou Clinical Research and Translational Center for Major Neurological Diseases (201604020010).

## Conflict of interest

The authors declare that the research was conducted in the absence of any commercial or financial relationships that could be construed as a potential conflict of interest.

## Publisher's note

All claims expressed in this article are solely those of the authors and do not necessarily represent those of their affiliated organizations, or those of the publisher, the editors and the reviewers. Any product that may be evaluated in this article, or claim that may be made by its manufacturer, is not guaranteed or endorsed by the publisher.
