# Corrigendum: Leflunomide combined with low-dose prednisone inhibits proinflammatory T cells responses in myasthenia gravis patients

**DOI:** 10.3389/fneur.2023.1179017

**Published:** 2023-03-15

**Authors:** Xin Huang, Hao Ran, Yingkai Li, Qian Ma, Changyi Ou, Li Qiu, Huiyu Feng, Weibin Liu

**Affiliations:** ^1^Department of Neurology, The First Affiliated Hospital, Sun Yat-sen University, Guangzhou, China; ^2^Guangdong Provincial Key Laboratory of Diagnosis and Treatment of Major Neurological Diseases, National Key Clinical Department and Key Discipline of Neurology, Guangzhou, China; ^3^School of Pharmaceutical Sciences, Sun Yat-sen University, Guangzhou, China

**Keywords:** myasthenia gravis, leflunomide, low dose prednisone, Th1 cells, Th2 cells, Th17 cells, Th9 cells

In the published article, an author name was incorrectly written as Yingkia Li. The correct spelling is Yingkai Li.

The authors apologize for this error and state that this does not change the scientific conclusions of the article in any way. The original article has been updated.

